# Characterization of myocardial fiber orientation to assess therapeutic exosomes from cardiosphere-derived cells (CDCs) in myocardial infarcted porcine with in vivo diffusion-tensor CMR on a clinical scanner

**DOI:** 10.1186/1532-429X-18-S1-Q62

**Published:** 2016-01-27

**Authors:** Christopher T Nguyen, James Dawkins, Elizabeth M Tunnicliffe, Xiaoming Bi, Matthew D Robson, Debiao Li, Eduardo Marbán

**Affiliations:** 1 grid.50956.3f0000000121529905Biomedical Imaging Research Institute, Cedars-Sinai Medical Center, Los Angeles, CA USA; 2 grid.50956.3f0000000121529905Heart Institute, Cedars-Sinai Medical Center, Los Angeles, CA USA; 3 grid.4991.50000000419368948Oxford Centre for Clinical Magnetic Resonance Research, Division of Cardiovascular Medicine, Radcliffe Department of Medicine, University of Oxford, Oxford, United Kingdom; 4 MR R&D, Siemens Healthcare, Los Angeles, CA USA; 5 grid.19006.3e0000000096326718Bioengineering, University of California Los Angeles, Los Angeles, CA USA

## Background

Diffusion-Tensor cardiovascular magnetic resonance (DT-CMR) is capable of mapping myocardial fiber orientation [1,2]. It has been demonstrated in myocardial infarction (MI) murine models that DT-CMR can identify the effects of stem cell therapy on myocardial fiber orientation [3]. However, it remains to be seen if this recent work is translatable to large animal and clinical studies. We propose the application of a well-established in vivo cardiac DT-CMR technique [4] to characterize myocardial fiber orientation before and after the novel regenerative therapy of using intramyocardial injection of exosomes from cardiosphere-derived cells (CDCs) in a MI porcine model.

## Methods

MI was induced in 7 Yucatan mini pigs by balloon occlusion of the mid-LAD for 2.5 hours. The MI was allowed to heal for 4 weeks for all pigs defining baseline. Group 1 (N = 4) were treated with exosome proteins derived from CDCs. Group 2 was given a placebo.

CMR was performed at baseline and 4 weeks after treatment on a 3T Siemens Verio with the following: whole-heart 2D multi-slice (WH) morphological CMR, WH function CINE CMR, 3 short axis slice DT-CMR (STEAM, 6 dir, b = 350 s/mm^2^, 8 avg, 2 avg/BH), and WH viability CMR (LGE PSIR, TI = 315 ms). Viability and function CMR yielded scar size (SS) and ejection fraction (EF) [5], respectively. For in vivo DT-CMR, mean diffusivity (MD), fractional anisotropy (FA), and helix angle (HA) maps were calculated. HA transmurality slope (HATS) was calculated by radially sampling the transmural HA along 36 chords and fitting the slope of a linear regression between HA and transmural depth.

Wilcoxon signed-rank test was performed to evaluate the difference between mean slice values (G1 N = 12, G2 N = 9) before and after treatment (p < 0.017 significance). Change (Δ) in MD, FA, and HATS were correlated (R^2) with ΔSS and ΔEF.

## Results

For Group 1 (treated), EF, MD, FA, and HATS did not significantly change (Δ: -1 ± 2%, -0.1 ± 0.2 um^2^/ms, 0.01 ± 0.03, and 0.05 ± 0.15°/%depth, respectively), while SS was significantly reduced (Δ: -3 ± 2%, p < 0.01). In contrast, Group 2 (placebo) exhibited significant (p < 0.01) adverse changes with decreased EF (Δ: -4 ± 2%), increased SS (Δ: 3 ± 1%), decreased FA (Δ: -0.03 ± 0.02), and less helical HATS (-1.2 ± 0.1 vs -0.9 ± 0.2°/%depth). ΔMD and ΔFA weakly correlated with ΔEF (R^2: 0.02 and 0.27, respectively) and ΔSS (R^2: 0.03 and 0.08, respectively). However, ΔHATS significantly (p < 0.01) correlated highly with ΔEF (R^2: 0.85) and ΔSS (R^2: 0.67).

## Conclusions

In a MI porcine model, in vivo DT-CMR revealed that myocardial fiber orientation was preserved with CDC-derived exosome treatment and adversely changed with placebo treatment consistent with observed viability and function changes. Furthermore, changes in helix transmurality highly correlated with changes in viability and function.

**Figure 1:**
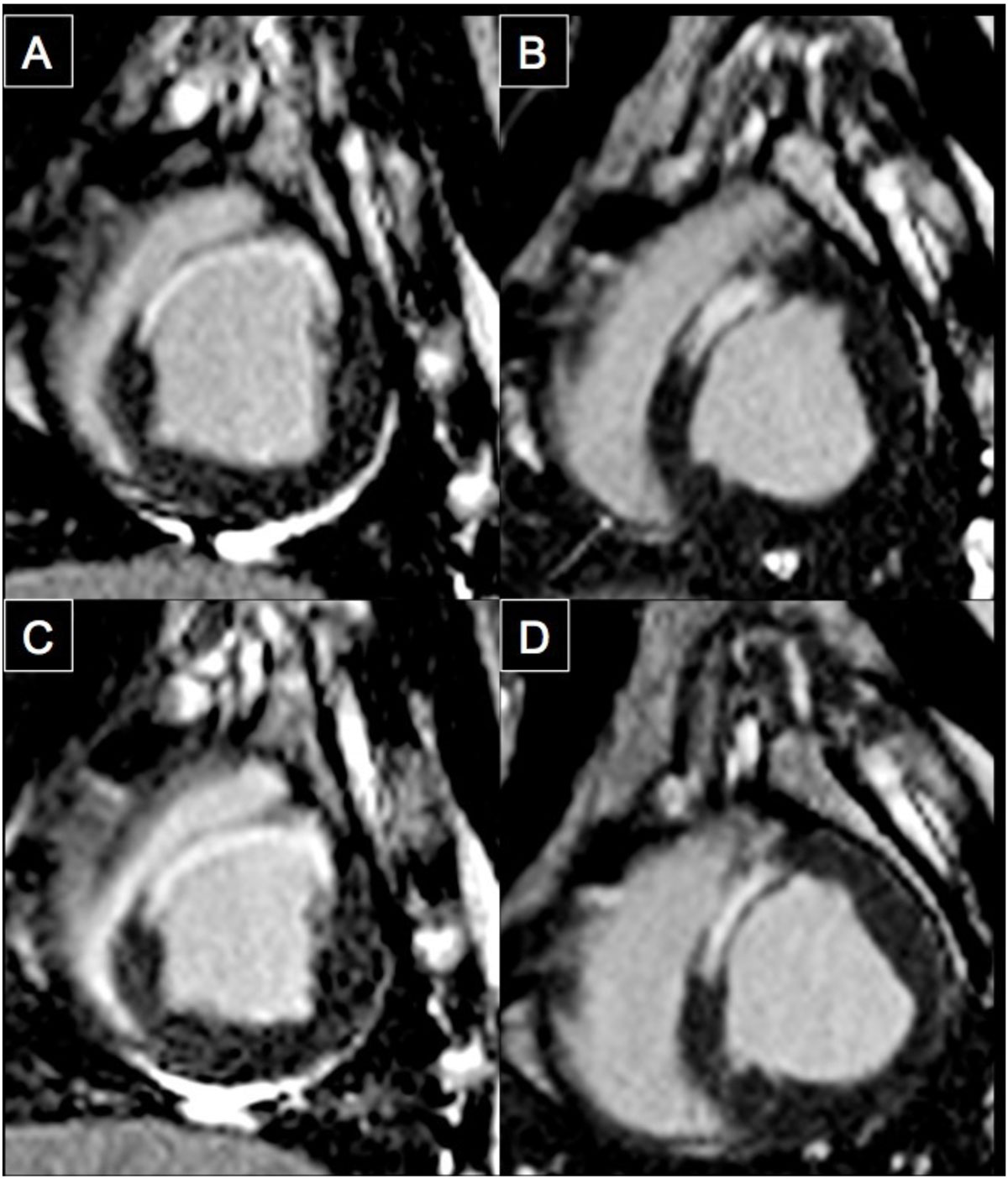
**Representative viability images of pre-treatment (A, B) and post-treatment with exosome (C) and placebo (D)**. Scar size is maintained (1% difference) with exosome treatment and greatly increased (10%) with placebo treatment.

**Figure 2:**
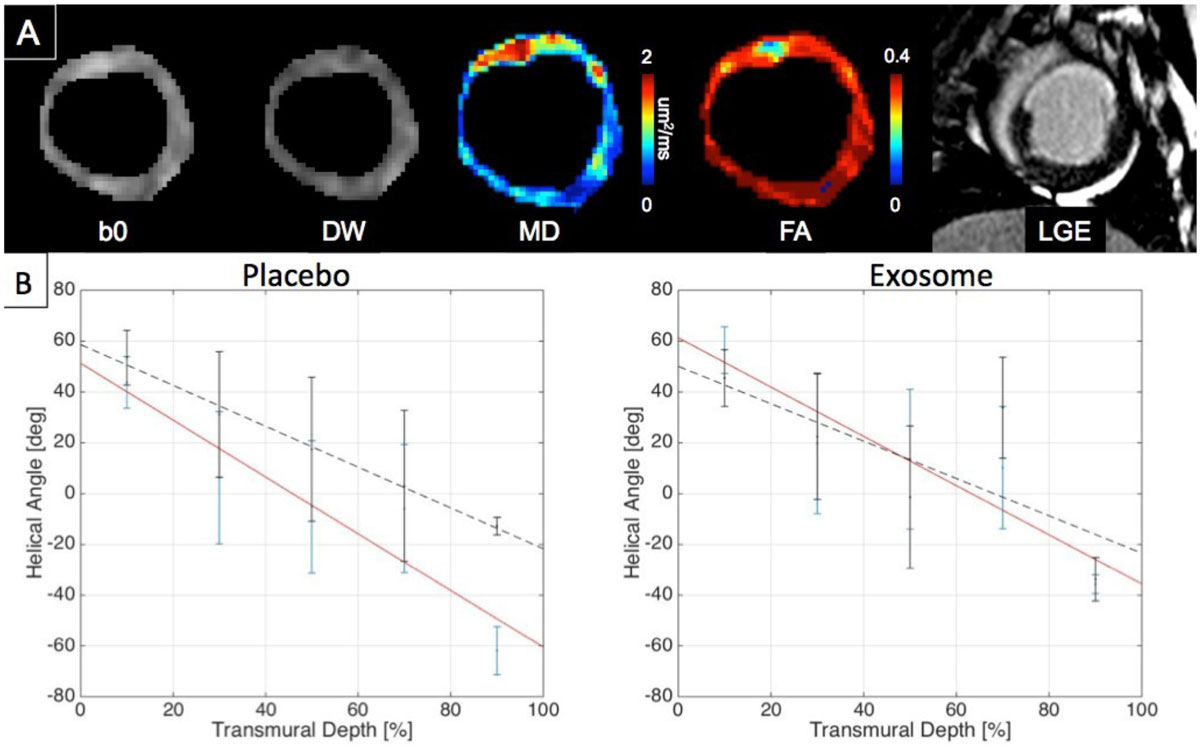
**Representative images (A) of least diffusion weighted (b0), diffusion weighted (DW), MD, FA, and LGE of a baseline measurement 4 weeks after MI**. Scar is demarcated with increases in MD, decreases in FA, and increase in LGE signal. Representative helix angle vs transmural depth plots (B) before (red line) and after (black dotted line) treatment. Note the placebo treatment causes the slope of the line to flatten out (slope à 0) indicating less helical transmurality, while exosome treatment maintains the helical transmurality.
